# Concurrent Blockade of Endothelial EGFR and VEGF Signaling on Malignant Associated Pleural Fluid Induced Angiogenesis: From Clinic to Bench

**DOI:** 10.3390/biomedicines9101327

**Published:** 2021-09-26

**Authors:** Wei-Teing Chen, Yu-Huei Lin, Chih-Ying Changchien, Ying Chen, Hsin-Han Chang, Wen-Chiuan Tsai, Hao-Chung Tsai, Chieh-Yung Wang, Ming-Sheng Shen, Li-Ting Cheng, Chen-Liang Tsai

**Affiliations:** 1 Division of Chest Medicine, Department of Medicine, Cheng-Hsin General Hospital, Taipei 112, Taiwan; stigma712@yahoo.com.tw; 2 Department of Internal Medicine, Tri-Service General Hospital, National Defense Medical Center, Taipei 114, Taiwan; koala8072@yahoo.com.tw; 3 Post-Baccalaureate Program in Nursing, College of Nursing, Taipei Medical University, Taipei 110, Taiwan; gracelin@tmu.edu.tw; 4 Department of Biology and Anatomy, National Defense Medical Center, Taipei 114, Taiwan; eva.flower@gmail.com (Y.C.); albertchang1008@gmail.com (H.-H.C.); 5 Department of Pathology, Tri-Service General Hospital, National Defense Medical Center, Taipei 114, Taiwan; ab95057@hotmail.com; 6 Division of Chest Medicine, Department of Internal Medicine, Tri-Service General Hospital Songshan Branch, Taipei 105, Taiwan; petstsai@yahoo.com.tw; 7 Division of Pulmonary and Critical Care Medicine, Department of Internal Medicine, Tri-Service General Hospital, National Defense Medical Center, Taipei 114, Taiwan; marechalparis@gmail.com (C.-Y.W.); letim47@gmail.com (L.-T.C.); 8 Department of Internal Medicine, Taichung Armed Force General Hospital, Taichung 411, Taiwan; darkevilalien@gmail.com

**Keywords:** lung cancer, malignant associated pleural effusion, vascular endothelium, EGFR, gefitinib, bevacizumab, exosome

## Abstract

Malignant-associated pleural fluid (MAPF) represented an unsolved problem in advanced lung cancer. Our previous work characterized increased pleural angiogenesis in lung adenocarcinoma and the propensity of MAPF on endothelial angiogenesis. This study investigated the combined efficacy of the tyrosine kinase inhibitor (gefitinib) and bevacizumab in opposing MAPF-induced angiogenesis. In lung adenocarcinoma patients with malignant pleural effusion (MPE), Kaplan–Meier analysis revealed the benefit of cotreatment with target therapy and bevacizumab. Increased EGFR expression was observed in the pleural microvessels of patients with lung adenocarcinoma both with and without mutations in EGFR. MAPF was obtained from lung adenocarcinoma patients both wild-type and mutant EGFRs. Total and phosphorylated EGFR were upregulated in HUVEC cultured with MAPF. Treatment with gefitinib as an EGFR inhibitor suppressed MAPF-induced endothelial migration and partially attenuated endothelial proliferation in both wild-type and mutant EGFR lung adenocarcinoma. Cotreatment with gefitinib and bevacizumab produced better inhibition of MAPF-induced endothelial angiogenesis than gefitinib alone in the mutant EGFR subgroup. Protein analysis of MAPF-derived exosomes revealed abundant EGFR and p-EGFR components that implied possible transfer to endothelial cells. Concluding Kaplan–Meier analysis and in vitro studies, the results indicated that the addition of bevacizumab on gefitinib treatment could suppress MAPF-induced angiogenesis in lung adenocarcinoma patients.

## 1. Introduction

Recurrent symptomatic MPE in non-small cell lung cancer (NSCLC) is a troublesome and unresolved clinical problem [1]. MPE is associated with excess lung fluid containing malignant cells and elevated lactate dehydrogenase (LDH) whether patients have actionable mutations or not [2]. The combination of epidermal growth factor receptor–tyrosine kinase inhibitors (EGFR-TKIs) with vascular endothelial growth factor (VEGF) blockade has been shown to produce an improved progression-free survival in patients with advanced lung cancer [3,4,5]. Moreover, 54% of patients with MPE were responsive to EGFR-TKI, and 74% of patients harboring an *EGFR* mutation were susceptible to EGFR-TKI [6]. Both EGFR and VEGFR2 are critical regulators in cancer progression, with the upregulation of VEGFA-VEGFR2 signaling pathways contributing to the resistance to EGFR-TKI treatment [7]. In surgical specimens, double immunofluorescence has revealed phosphorylated (p-EGFR) and p-VEGFR upregulation in tumor endothelial cells [8]. In endothelial cells isolated from EGFR-TKI treated tumors, increased VEGFR2 mRNA and protein expression, suggesting heightened sensitivity to the inhibitor [9]. However, the efficacy of combined EGFR and VEGF blockade was not addressed in lung cancer patients complicated with MPE, particularly in pleural endothelial cells.

We have reported increased pleural angiogenesis in patients with both lung and breast cancer [10,11]. Furthermore, in patients with lung cancer, MPE and paramalignant pleural effusion (PPE) were integrated as malignant-associated pleural fluid (MAPF). MAPF has been shown to stimulate endothelial proliferation, migration, and angiogenesis. There are shared characteristics between tumor endothelial cells and human umbilical vein endothelial cells (HUVECs) cultured with MAPF, including increased mitosis, filopodia formation, and aberrant angiogenesis [12,13]; the cancerous cells stimulate endothelial cells to increase EGFR expression through VEGF-related paracrine effects [14]; the cancerous cells stimulate endothelial cells to increase EGFR expression through VEGF-related paracrine effects [15]. In addition, there is intercellular EGFR protein transfer from tumor-derived exosomes to endothelial cells [16]. Distinct from their normal counterparts, tumor endothelial cells are more responsive to EGF incubation with activation of mitogen-activated protein kinase (MAPK) signaling pathways and increased proliferation (14). In a melanoma xenograft model, cancer cells expressed very little EGFR, and targeting endothelial EGFR with TKI sufficiently retarded tumor growth [9]. However, the role of EGFR signaling on pleural endothelial cells and the potency of EGFR-TKI in alleviating MAPF-induced angiogenesis remains undetermined.

Our previous study showed increased VEGFR2 expression in HUVEC cultured with MAPF, and the application of a VEGF blockade attenuated MAPF-induced endothelial angiogenesis. A promising TKI (gefitinib) and an anti-VEGF-A antibody (bevacizumab) have been approved for clinical use. However, the combined effect of these drugs on the inhibition of MAPF-induced endothelial angiogenesis has not been thoroughly tested. Moreover, tumor-cell-released exosomes were found to promote angiogenesis through an intercellular transfer of DNA, RNA, and oncogenic protein [17]. In lung cancer, intercellular communication through exosomes has been found to contribute to the resistance of EGFR-TKI and anti-angiogenesis therapy [18,19]. The characterization of exosomes in promoting MAPF-induced angiogenesis has not been investigated before. Accordingly, the present in vitro study aims to investigate the role of MAPF-induced endothelial EGFR expression and the efficacy of combined VEGF blockade with bevacizumab and gefitinib to suppress MAPF-induced angiogenesis in patients with lung cancer harboring mutant or wild-type EGFR using HUVECs.

## 2. Materials and Methods

### 2.1. Kaplan–Meier Analysis of Lung Adenocarcinoma Patients

**Figure 1:**
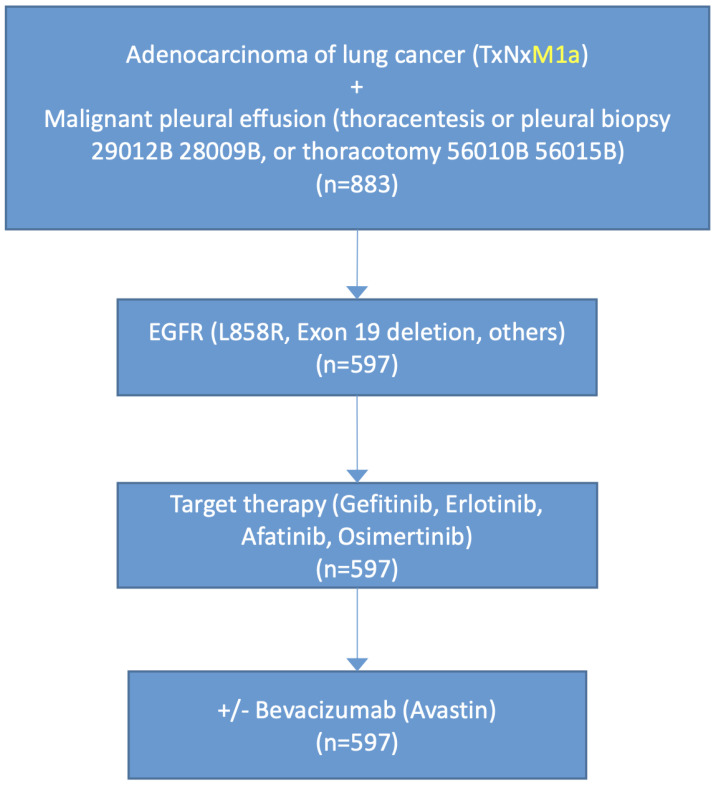
Flow diagram illustrating case selection of lung adenocarcinoma patients. TxNxM1a indicates stage IV lung cancer with lung or pleural metastasis no mater tumor size or lymph node metastasis status.

We gathered clinical data from Taipei Medical University Institutional and Clinical Database, which contains patients’ medical information for 3 million patients who visited Taipei Medical University Hospital, Wan Fang Hospital, and Shuang Ho Hospital (IRB number-TSGH No. B202005001, 19 February 2020 and TMU-JIRB NO. N202102071, 20 February 2021). We retrospectively reviewed the stage IV NSCLC patients from January 2010 to December 2019 to validate the outcome. Those included met the following criteria illustrate in Figure 1: (1) age 30 or over; (2) pathologic diagnosed with adenocarcinoma of lung; (3) presented MPE; (4) underwent target therapy (EGFR-TKI). The study outcome was defined as time to encountered chemotherapy or survival.

### 2.2. Patient Characteristics and Collection of Pleural Fluid Samples

The study was approved by the Institutional review board (IRB) of the Tri-Service General Hospital (TSGH) Research Ethics Committee. Under sonography-guided thoracentesis, pleural fluid samples were obtained from patients with lung cancer who provided written informed consent. Patient data were extracted from their medical records including cancer staging, demographics, staging, treatment regimens, and *EGFR* mutation status (Table 1). A total of 5 mL of MAPF was collected from each patient. Fresh samples were immediately centrifuged at 1000× *g* for 15 min and filtered (0.22 µm, Millipore, Darmstadt, Germany) to obtain a cell-free specimen. All samples were stored at −80 °C and thawed once before use.

### 2.3. Hematoxylin and Eosin (HE) Staining and Immunohistochemistry

Pleural tissues were fixed in 10% *v*/*v* formalin, embedded in paraffin, and sectioned at 6 μm on a microtome. The paraffin sections were deparaffinized and stained with HE in a standard manner to assess general tissue morphology. For immunohistochemical staining, pleural tissues were fixed in phosphate-buffered saline (PBS; 137 mM NaCl, 2.7 mM KCl, 1.5 mM KH_2_PO_4_, and 8 mM Na_2_HPO_4_ pH 7.4), with 10% *v*/*v* formaldehyde, 4% *w*/*v* sucrose, and 0.15 mM CaCl_2_, incubated with permeabilization buffer (PBS with 0.2% *v*/*v* Triton X-100, or PBST) and blocked with blocking buffer (PBST with 5% *w*/*v* nonfat milk). After antigen retrieval, pleural tissues were incubated with primary antibody against rabbit EGFR (1:200 dilution, Millipore) and secondary goat anti-rabbit antibody (Jackson ImmunoResearch Laboratories, Inc., West Grove, PA, USA).

### 2.4. Culture of Primary Endothelial Cells

HUVECs were purchased from The Bioresource Collection and Research Center (BCRC, Hsinchu, Taiwan) and cultured in an endothelial cell medium (ScienCell Research Laboratories, San Diego, CA, USA). In MAPF treated group, HUVEC were cultured with 30% MAPF (*v*/*v*) for indicated hours.

### 2.5. Drugs and Reagents

Dimethyl sulfoxide (DMSO), 3-(4,5-dimethylthiazol-2-yl)-2,5-diphenyltetrazolium bromide (MTT), and Coomassie brilliant blue G-250 were purchased from Sigma-Aldrich (St. Louis, MO, USA). Gefitinib, marketed as Iressa, was obtained from AstraZeneca and dissolved in DMSO. Bevacizumab, marketed as Avastin, was obtained from Roche.

### 2.6. Cell Survival Assay

HUVECs were plated at a density of 2 × 10^4^ per well in a 96-well plate. MAPF was then added to the culture medium with 30% *v*/*v* and incubated for 24 h. After the cells were washed with PBS, 0.5 mg/mL MTT was added and the plates were incubated for another 4 h. Cells were then lysed with DMSO. The absorbance was measured at 590 nm for each well. All results were calculated from six independent experiments.

### 2.7. Transwell Assays

HUVECs were seeded in the upper chamber of a Transwell^®^ plate (Corning Costar, USA) at a density of 2 × 10^4^ per well. After treatment with MAPF for 16 h, those that migrated to the lower chamber were fixed with 10% *v*/*v* formalin, washed with PBS, and stained with Coomassie brilliant blue G-250. The migrated cells were examined in three randomly selected fields from each membrane in six independent experiments.

### 2.8. Tube Formation Assay

The 96-well plates were prechilled, then 50 μL of Matrigel (Corning Costar, USA) was added to each well and incubated for 1 h at 37 °C. Then, HUVECs were seeded at a density of 1 × 10^4^ cells per mL of medium containing 30% MAPF (*v*/*v*). After a 12 h incubation, tube formation was photographed, and the widths of capillary networks were quantified using ImageJ. All results were calculated from six independent experiments.

### 2.9. Western Blotting

HUVECs were rinsed once with PBS and lysed with 60 mM PIPES (piperazine-N,N′-bis (2-ethanesulfonic acid)), 25 mM HEPES (N-(2-hydroxyethyl)piperazine-N′-(2-ethanesulfonic acid), 0.15% Triton X-100, 10 mM ethylene glycol-bis(β-aminoethyl ether)-N,N,N′,N′-tetraacetic acid, 2 mM magnesium chloride, 1 mM sodium fluoride, 2.5 mM sodium pyrophosphate, 1 mM phenylmethylsulfonyl fluoride, 1 mM sodium orthovanadate, 1 mM β-glycerophosphate, 1 μg/mL leupeptin, 1 μg/mL pepstatin A, and 1 μg/mL aprotinin (pH 6.9). 40µg of each sample were separated by 10% sodium dodecyl sulfate–polyacrylamide gel electrophoresis (SDS-PAGE), then proteins were transferred to a nitrocellulose membrane (Bio-Rad) according to the manufacturer’s instructions. The membranes were incubated overnight at 4 °C with primary antibodies in Tris-buffered saline with Tween (TBST) (50 mM Tris-HCl, 150 mM sodium chloride, and 0.1% *v*/*v* Tween-20, pH 7.4). The primary antibodies were specific for glyceraldehyde 3-phosphate dehydrogenase (GAPDH, Cell Signaling Technology, Danvers, MA, USA), EGFR (Millipore, USA), and p-EGFR (Cell Signaling Technology, USA). After the membranes were washed, the strips were incubated with a 1:5000 or 1:10000 dilution of horseradish peroxidase-conjugated anti-rabbit IgG from Cell Signaling Technology, USA. Next, the blots were treated with a chemiluminescent substrate developing solution (Bio-Rad, Hercules, CA, USA). Band densities were captured and quantified by densitometry using ImageJ. The control sample was set as 100%, and the test samples were normalized to the control. All MAPF samples were cocultured with HUVEC cells and examined the expression of EGFR and p-EGFR levels.

### 2.10. Immunofluorescence Staining

HUVECs were seeded on coverslips and incubated in the presence of MAPF for 8 h. Cells were then rinsed with PBS and fixed with 10% *v*/*v* formalin in PBS (pH 7.4). A blocking solution (5% milk in 0.1% *v*/*v* Triton X-100) was applied to prevent nonspecific binding. Primary antibody against VE-cadherin (Cell Signaling Technology, USA) and β-catenin (BD Bioscience, USA) in blocking buffer was incubated with the HUVECs at 4 °C overnight. After the antibody was washed, the slides were incubated with fluorescein isothiocyanate-conjugated goat anti-mouse and anti-rabbit IgG (Sigma-Aldrich) for 1 h. Finally, coverslips were mounted with mounting medium (Gel Mount Aqueous, Sigma) and photographed with a Nikon D1X digital camera (Carl Zeiss, Oberkochen, Germany).

### 2.11. Vascular Permeability Assay

In vitro assays were performed according to the manufacturer’s protocol (Merck Millipore, Darmstadt, Germany). When HUVEC cells reached confluency on a transwell insert, the upper chambers were replaced with MAPF, gefitinib, and bevacizumab, as stated in the figure legends. Following induction of permeability, the medium in the upper chamber was reconstituted with a fluorescein isothiocyanate (FITC)–Dextran solution. 100 µL of the media from each well of the receiver tray was collected, and the fluorescence was measured with a plate reader using excitation and emission at 485 and 535 nm, respectively. All results were calculated from six independent experiments.

### 2.12. Exosome Purification

Exosomes were isolated from 10 mL of 0.22 µm filtrated pulmonary pleural effusion using an ultracentrifugation protocol from the International Society for Extracellular Vesicles (ISEV) [20]. Isolated exosomes were characterized using nanoparticle tracking analysis by Nanosight (NanoSight NS300, Malvern Panalytical). Representative images were shown in figures.

### 2.13. Transmission Electron Microscopy (TEM)

Isolated exosomes were fixed in 2% paraformaldehyde/1% glutaraldehyde. The fixed nanoparticles were absorbed to formvar-coated copper grids for 20 min. After being washed, the grids were stained with 2% uranyl acetate. Exosomes’ morphology was visualized with TEM (Hitachi HT7700 microscope, Hitachi High-Technologies Corporation), and images were recorded at 75 kV.

### 2.14. Statistical Analysis

Data are expressed as the averages of at least triplicate samples and are presented as the mean ± standard error of the mean (SEM). Analysis was performed using Student’s *t*-test, with a *p*-value of < 0.05 chosen to indicate statistical significance. All results were calculated from six independent experiments.

## 3. Results

### 3.1. Clinical Response of Combined Target Therapy and Bevacizumab in Patients with MPE

**Figure 2:**
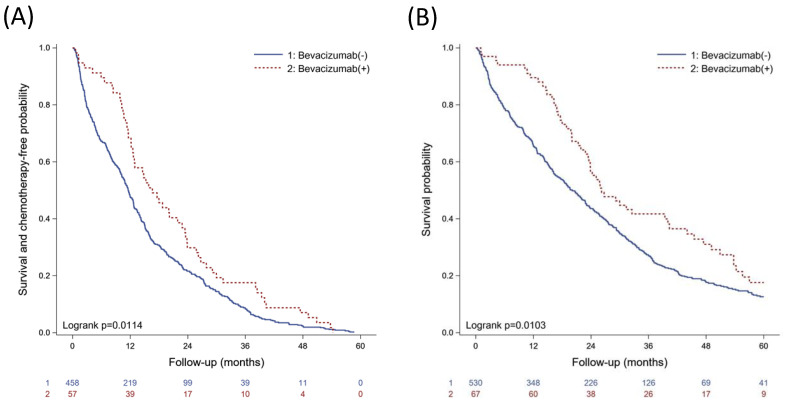
Kaplan–Meier analysis of lung adenocarcinoma patients with MPEl (**A**) survival and chemotherapy-free probability; (**B**) mortality.

In total, 883 lung adenocarcinoma patients complicated with MPE were enrolled for analysis. In the study population, 597 patients had *EGFR* mutations and received target therapy. Patients were divided into two groups based on whether they received the addition of bevacizumab therapy. A total of 67 patients underwent target therapy combination with bevacizumab, and 530 patients were on target therapy alone. The patient characteristics are listed in Supplementary Table S1. From Kaplan–Meier analysis, patients receiving cotreatment of target therapy and bevacizumab showed prolonged chemotherapy-free period and survival (Figure 2). Accordingly, combined target therapy and anti-angiogenesis agents could benefit lung adenocarcinoma patients with MPE.

### 3.2. Upregulated EGFR Expression in Pleural Capillaries in Lung Adenocarcinomas

**Figure 3:**
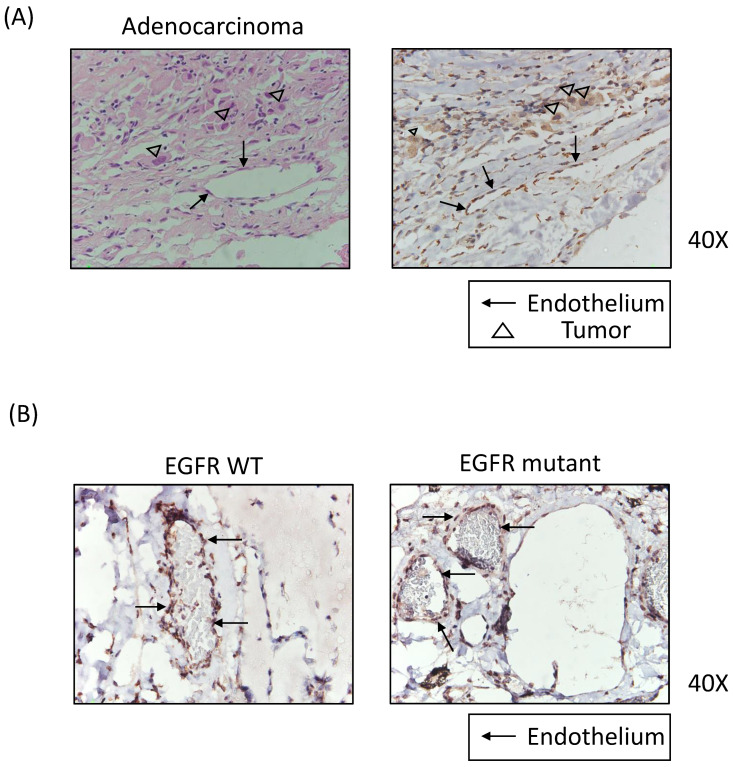
Histology and immunohistochemistry of pleural microvessels of lung adenocarcinoma: (**A**) hema-toxylin and eosin staining of pleura tissue of lung adenocarcinoma and the corresponding section of EGFR immunostaining; (**B**) representative images of pleural microvessel stained positively for EGFR. Lung adenocarcinoma specimens of wild-type (WT) EGFR and mutant EGFR were demon-strated in the left and right panels, respectively. (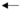, endothelial cell; 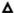, cancer cell) The magni-fication is 400x.

In pleural tissues of EGFR-mutated lung adenocarcinoma, histology revealed microvessel formation (Figure 3A) similar to that previously reported [10]. Corresponding immunohistochemistry sections had positive EGFR staining in both tumor cells and endothelial cells. Both wild-type and mutated EGFR lung adenocarcinoma specimens had enhanced expression of EGFR in pleural endothelial cells (Figure 3B). In addition, pleural endothelium samples were positive for EGFR in squamous cell carcinoma samples (Figure S1). Therefore, increased endothelial EGFR expression is accompanied by pleural angiogenesis in lung cancer.

### 3.3. MAPF-Induced Endothelial EGFR Upregulation and Gefitinib Treatment on MAPF-Induced Endothelial Proliferation and Migration

**Figure 4:**
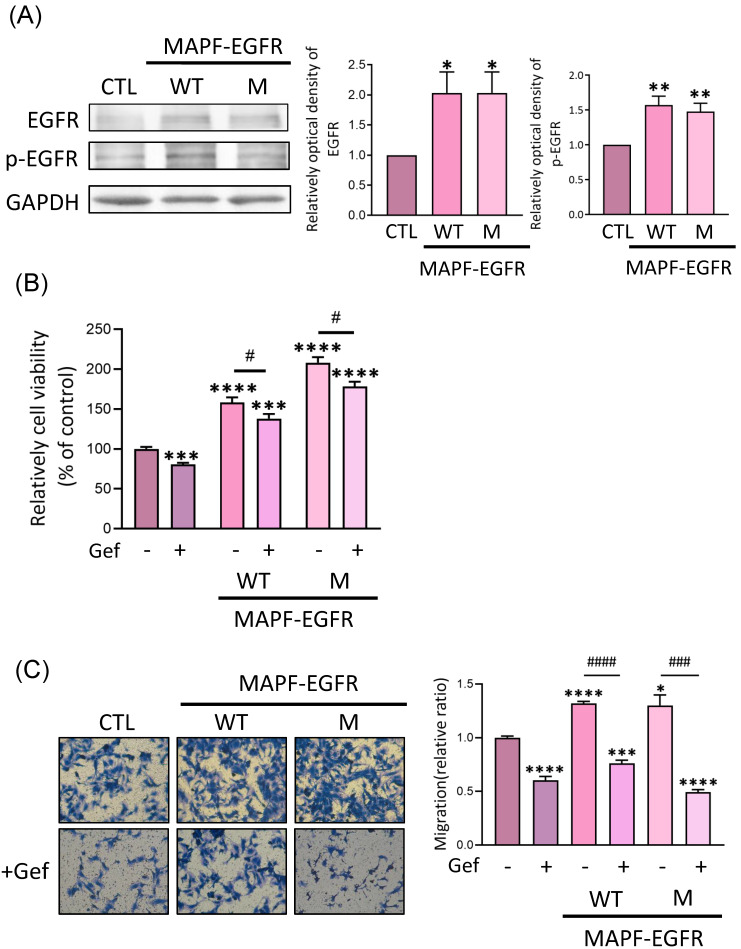
MAPF-induced endothelial EGFR upregulation and gefitinib treatment to ameliorate MAPF-induced endothelial migration. MAPF from patients with wild-type (WT) and mutant (M) EGFRs were analyzed separately: (**A**) HUVECs incubated with MAPF or control medium for 24 h. EGFR and p-EGFR protein expression were examined by Western blotting. GAPDH was used as an internal control; (**B**,**C**) HUVECs were incubated with MAPF in the presence or absence of 20 µM gefitinib for the indicated times. Cell viability was analyzed by MTT assay after 24 h of treatment. * *p* < 0.05; ** *p* < 0.01; *** *p* < 0.005; **** *p* < 0.0001 compared to the control group. # *p* < 0.05; ### *p* < 0.001; #### *p* < 0.0005, compared to the MAPF group. CTL stands for control. Gef stands for gefitinib.

We next examined the effect of MAPF on total and phosphorylated EGFR expression in HUVECs. Elevated EGFR and p-EGFR protein levels were found in HUVEC cultured with MAPF from wild-type and mutant EGFR samples (Figure 4A). An EGFR-TKI (gefitinib) was applied to evaluate the effect of EGFR blockade on MAPF-induced endothelial viability and motility. In the MTT assay, cotreatment with 20 μmol/L gefitinib for 24 h suppressed MAPF-induced endothelial viability around 20% in both wild-type and mutant EGFR samples (Figure 4B). Titration of gefitinib up to 1 μmol/L showed an inhibitory effect of MAPF-induced endothelial viability only when EGFR had an L858R mutation, with the exon 19 deletion/T790M mutation unresponsive (Figure S2). In the Transwell assay, the EGFR inhibitor suppressed MAPF-induced endothelial migration in both wild-type and mutant EGFR samples (Figure 4C). The above results suggested that gefitinib treatment as an EGFR inhibitor counteracted endothelial migration by MAPF.

### 3.4. Inhibitory Effect of Gefitinib Plus Bevacizumab on MAPF-Upregulated Endothelial Viability, Permeability, and Angiogenesis

**Figure 5:**
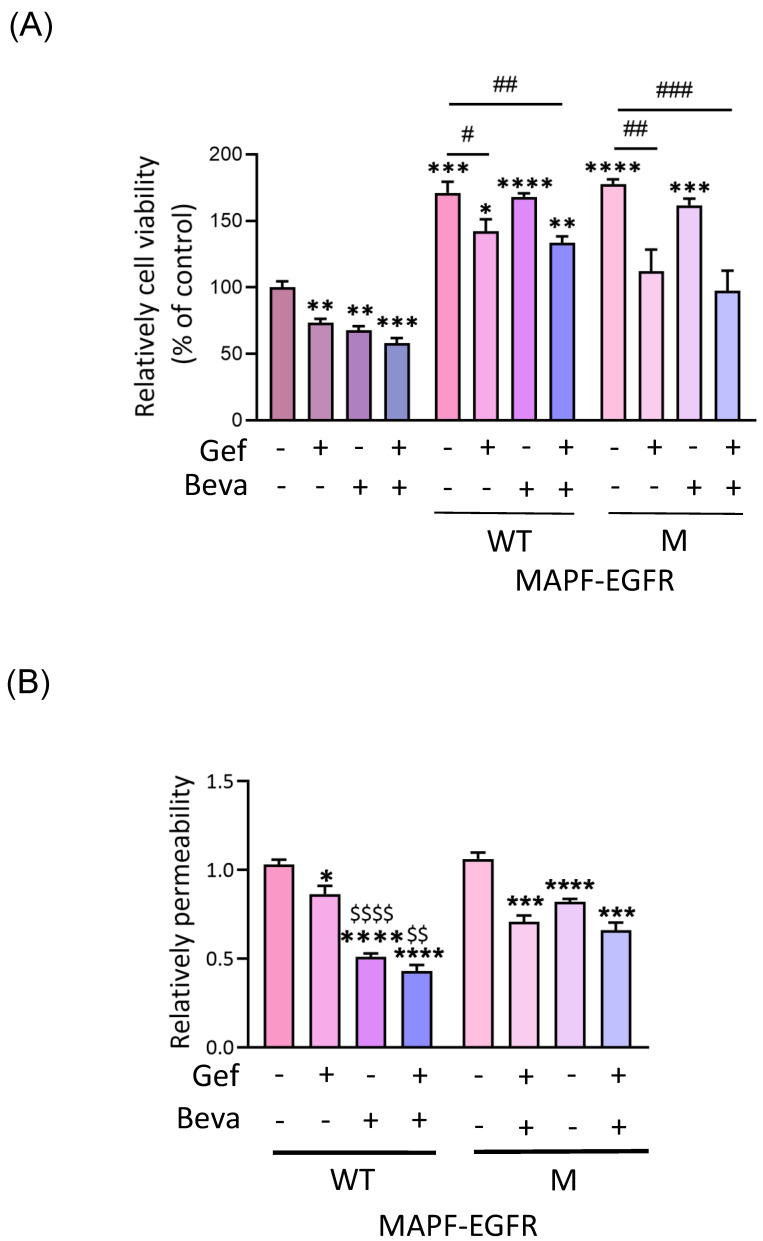
Gefitinib with bevacizumab alters MAPF-induced endothelial viability and permeability. HUVEC cells were treated with gefitinib (Gef), bevacizumab (Beva), or Gef/Beva in combination, in MAPF-containing medium for the indicated times. MAPF from patients with wild-type (WT) or mutant (M) EGFRs were analyzed separately. Concentrations of gefitinib and bevacizumab were 20 µM and 0.01 mg/mL, respectively: (**A**) cell viability was analyzed by MTT assay after 24 h of treatment; (**B**) endothelial permeability was determined by transwell permeability assay after 18 h of treatment. * *p* < 0.05; ** *p* < 0.01; *** *p* < 0.001; **** *p* < 0.0001, compared to the control group. # *p* < 0.05; ## *p* < 0.01; ### *p* < 0.001, compared to the MAPF group. $$ *p* < 0.01; $$$$ *p* < 0.0001 compared to the gefitinib-treated group.

Our previous study demonstrated the ability of the anti-VEGF-A antibody (bevacizumab) to attenuate MAPF-induced endothelial migration and angiogenesis. Therefore, we investigated the combined efficacy of gefitinib and bevacizumab in terms of MAPF-induced endothelial proliferation, permeability, and angiogenesis in HUVECs. MTT assays showed that the effect of bevacizumab on MAPF-induced endothelial proliferation was negligible in either wild-type EGFR or the mutant subgroup (Figure 5A). Endothelial proliferation induced by MAPF in the wild-type EGFR and mutant subgroup was suppressed around 20% by gefitinib 20 μM alone and with bevacizumab.

In cell permeability assay, there was decreased fluorescence intensity measured in MAPF-treated HUVEC when adding gefitinib, bevacizumab, or their combination (Figure 3B). Due to MAPF-induced increased cell numbers, there was a decreased permeability in the MAPF-treated group, compared with the control (Figure S3A). Both gefitinib and bevacizumab could mitigate endothelial cell permeability compared with the control (Figure S3B). The combination of gefitinib and bevacizumab demonstrated better inhibition of MAPF from the wild-type EGFR subgroup than gefitinib alone. The above results showed that combined gefitinib and bevacizumab could restore endothelial permeability in HUVEC treated with MAPF from wild-type and mutant EGFR groups.

**Figure 6:**
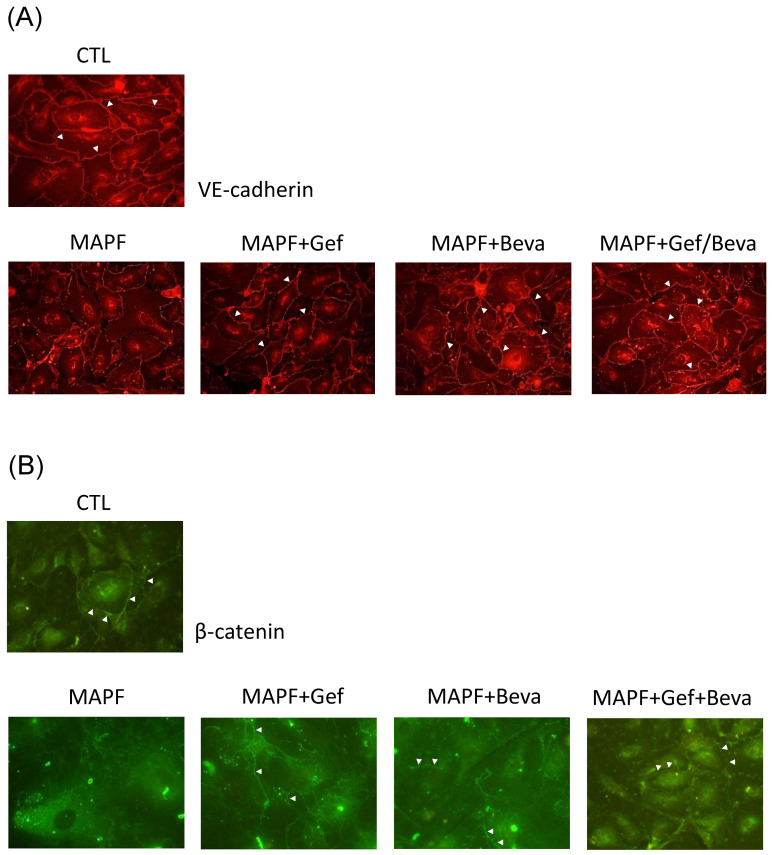
Gefitinib and bevacizumab alter MAPF-induced distribution of endothelial adherens junction proteins. HUVEC cells were treated with gefitinib (Gef), bevacizumab (Beva), or a Gef/Beva combination, in a MAPF-containing medium. CTL stands for control. MAPF was obtained from patients with *EGFR* mutations. Concentrations of gefitinib and bevacizumab were 20 µM and 0.01 mg/mL, respectively. After 12 h culture, the cells were subjected to immunofluorescence staining for VE-cadherin (**A**) and β-catenin (**B**). Arrowheads indicate distributions of junctional proteins between adjacent endothelial cells.

VE-cadherin and β-catenin are fundamental endothelial barrier proteins that regulate vessel permeability. Immunofluorescence staining was applied to examine VE-cadherin and β-catenin distribution in HUVEC cultured with MAPF from wild-type and mutant EGFR samples (Figure 6). In the wild-type, both VE-cadherin and β-catenin were distributed evenly at the cellular periphery, indicative of intact adherens junctions. After 12 h MAPF treatment, there was a loss of VE-cadherin and β-catenin localization at cell–cell contacts, implying a loosening of cell contacts. Treatment with gefitinib partially ameliorated MAPF-induced redistribution of VE-cadherin and β-catenin. The same effect was observed in HUVEC treated with MAPF and bevacizumab. Combined treatment of gefitinib and bevacizumab compensated for the MAPF effect, restoring endothelial barriers similar to untreated HUVEC cells.

**Figure 7:**
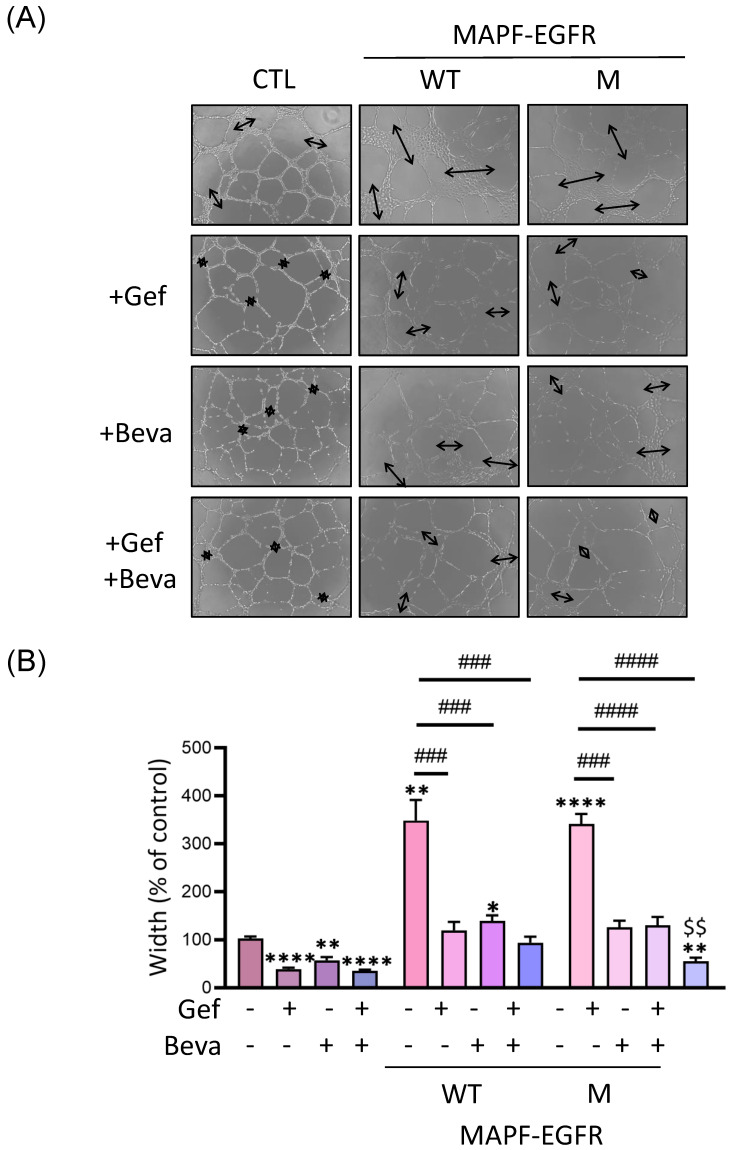
Gefitinib and bevacizumab alleviate MAPF-induced endothelial angiogenesis. HUVEC cells were treated with gefitinib (Gef), bevacizumab (Beva), or Gef/Beva in combination, in MAPF-containing medium for 12 h. MAPF from patients with wild-type (W) and mutant (M) EGFRs were analyzed separately. Concentrations of gefitinib and bevacizumab were 20 µM and 0.01 mg/mL, respectively: (**A**) representative images of tube formation after HUVEC cells cultured with above conditions for 12 h; (**B**) image analysis of tube width at 12 h. 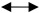 stands for tube width. * *p* < 0.05; ** *p* < 0.01; **** *p* < 0.0001, compared to the control group. ### *p* < 0.001; #### *p* < 0.0001, compared to the MAPF group. $$ *p* < 0.01, compared to the gefitinib-treated group.

In the tube formation assay, both gefitinib and bevacizumab attenuated the tube width increment by MAPF (Figure 7). A combined treatment of MAPF with gefitinib and bevacizumab demonstrated better suppression of the tubular width in the mutant EGFR. Therefore, either gefitinib alone, or gefitinib with bevacizumab modulates MAPF-induced vascular permeability and angiogenesis in wild-type and mutant EGFR samples.

### 3.5. Characterization of Exosome-Delivered EGFR, p-EGFR, and VEGFA in MAPF

**Figure 8:**
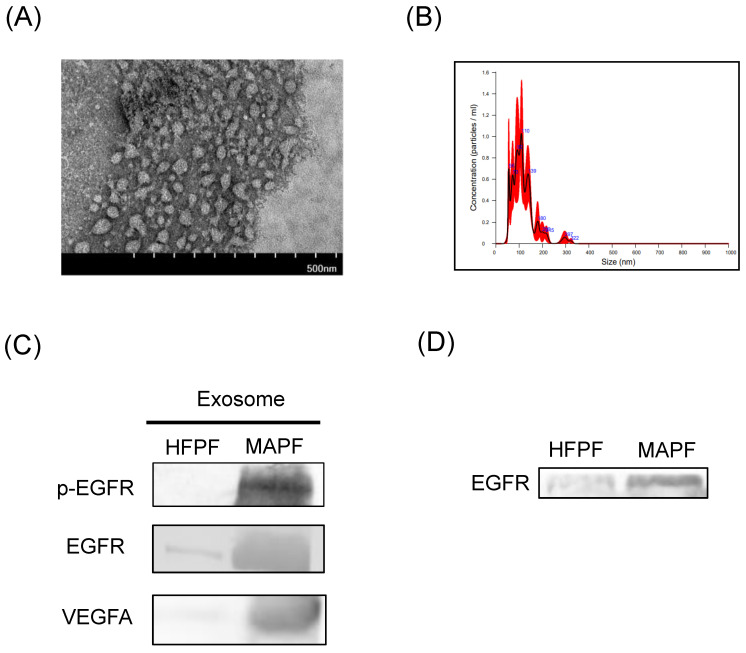
Increased expression of EGFR, p-EGFR, and VEGFA in exosomes isolated from MAPF: (**A**) a representative image of TEM showed exosomes derived from MAPF, scale bar = 500 nm; (**B**) the nanoparticle tracking analysis showed size distribution of exosomes derived from MAPF; (**C**) representative blot of p-EGFR, EGFR, and VEGFA expression in HFPF and MAPF-derived exosomes, respectively; (**D**) Western blot showed EGFR expression in MAPF.

Tumor-cell-derived exosomes containing EGFR were found to induce the angiogenic response of endothelial cells [16]. The investigation of extracellular vesicles and particles demonstrated the presence of exosomes in both heart-failure-associated pleural fluid (HFPF) and MAPF (Figure 8A). The mean exosome size was 129.5 ± 2.5 nm (Figure 8B). Compared with HFPF, the protein content of p-EGFR, EGFR, and VEGFA were more abundant in exosomes derived from MAPF (Figure 8C). EGFR expression was significantly higher in MAPF than HFPF. The expression of p-EGFR was undetectable in both HFPF and MAPF (Figure 8D). The above results might imply the mechanism of endothelial EGFR upregulation through EGFR transfer from MAPF-derived exosomes.

## 4. Discussion

Previous meta-analysis studies confirmed the dual pathway inhibitors had a superior effect on progression-free survival than EGFR-TKIs alone in advanced NSCLC [21,22]. Our study provided a real-world treatment response of combination EGFR TKI and anti-angiogenesis in adenocarcinoma of the lung with MPE. In resected NSCLC, elevated EGFR expression in tumors results in greater microvessel density [23]. Despite heterogeneity in IHC interpretation, tumor with EGFR overexpression was correlated with shorter survival in NSCLC [24,25]. Consequently, EGFR expression in pleural microvessels implies the angiogenic activity of MAPF and an unfavorable prognosis for patients with lung cancer. We characterized an association between pleural angiogenesis and endothelial EGFR expression from lung adenocarcinoma patients with MAPF; it also presented in the pleural tissue of squamous cell carcinoma.

To recapitulate the findings of EGFR expression in pleural endothelial cells, we cultured HUVEC with MAPF from lung cancer patients receiving thoracocentesis. A total of 15 lung cancer patients were recruited with 7 cases of wild-type EGFR and 8 cases with *EGFR* mutations (Table 1). Both MAPF from wild-type and mutant EGFRs upregulated endothelial EGFR and p-EGFR expression. In multiple tumor types, activation of EGFR signaling is often accompanied by the reproduction of angiogenic factors, including VEGF [26]. A blockade of EGFR activation significantly ameliorates tumor-induced angiogenesis [27]. Our characterization of MAPF-induced endothelial EGFR expression indicates that EGFR signaling plays a role in pleural angiogenesis.

Exosome-associated VEGFs were reported to be insensitive to bevacizumab that induced cancer progression in patients receiving bevacizumab therapy [28]. Our findings of exosomes containing VEGF could be correlated with the limitation of bevacizumab on MAPF-induced endothelial angiogenesis and suggest the need for additional signaling blockade. The mechanism of EGFR upregulation in tumor endothelial cells could be attributed to EGF secreted by cancer cells [14]. However, previous research did not report the abundance of EGF in MAPF that was compatible with our preliminaries (data not shown). Previous research showed exosomes released from lung cancer altered permeability and angiogenesis of endothelial cells [29,30]. Moreover, Khalid et al. published that activated EGFR transported by tumor-derived exosome could be uptake by endothelial cells and induced endothelial VEGF expression [16]. Our characterization of EGFR and p-EGFR in MAPF-extracted exosome could elaborate the mechanism of endothelial EGFR upregulation in MAPF culture.

The responsiveness of EGFR-TKI in pleural endothelial cells was investigated by cotreating HUVEC with MAPF and gefitinib. Gefitinib treatment downgraded MAPF-induced endothelial migration in patients with or without *EGFR* mutations. It has been reported that a minority of patients with wild-type EGFR respond to EGFR-TKI therapy in terms of tumor recurrence [6,31]. Our results further demonstrated the efficacy of EGFR-TKI to counteract pleural angiogenesis even in patients with wild-type EGFR. In colon cancer, EGFR activation of tumor endothelial cells is a crucial factor for determining the susceptibility of tumor therapy by EGFR-TKI [32]. For patients with EGFR gene mutations resulting in its increased expression, EGFR-TKI ameliorates the progression of malignant pleural effusion [33]. Treatment of gefitinib on MAPF-induced endothelial migration in this work further revealed the therapeutic mechanism of EGFR-TKI by targeting pleural microvessels.

Our previous study demonstrated the efficacy of bevacizumab in MAPF-induced endothelial migration and angiogenesis but not proliferation [10]. The current study of a combination of gefitinib with bevacizumab showed better inhibition of MAPF-induced endothelial permeability and angiogenesis in wild-type and mutant EGFR samples. These findings were in accordance with several clinical trials showing that anti-VEGF and EGFR combination therapy improves the overall prognosis in female patients with EGFR exon 19 deletion groups [34]. Furthermore, our results of MAPF from the wild-type subgroup were in line with results showing that bevacizumab therapy was effective in treating NSCLC patients with uncontrolled MPE and wild-type EGFR [35]. Therefore, simultaneous targeting of both VEGF and EGFR suppresses the MAPF-induced permeability and angiogenesis effects independent of the EGFR status in vitro.

## 5. Conclusions

**Figure 9:**
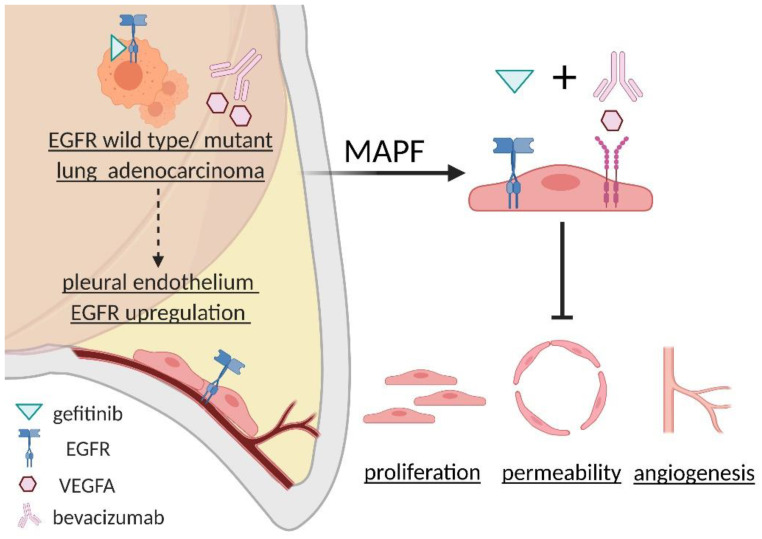
Scheme of MAPF-induced endothelial EGFR upregulation and synergetic blockade of EGFR and VEGFR on MAPF-induced endothelial angiogenesis.

The addition of anti-angiogenesis prolonged the EGFR target therapy treatment duration and prevented the patients from undergoing chemotherapy. EGFR expression on pleural endothelial cells provided a potential pathway that can be targeted to control the progression of lung cancer complicated with MAPF. The characterization of EGFR enriched exosomes in MAPF provided a possible mechanism for EGFR upregulation in HUVEC. In our subsequent in vitro study, we further explored combined gefitinib with bevacizumab significantly ameliorated MAPF-induced endothelial angiogenesis independent of EGFR status of lung adenocarcinoma patients (Figure 9).

## Figures and Tables

**Table 1:** Characteristics of lung adenocarcinoma patients complicated with MAPF.

	Total 19 (100%)
Numbers	19
Age, median, years	66.6 ± 10.8
Male	13 (68.4%)
Stage IV	19 (100%)
Histology	
Adenocarcinoma	18 (94.7%)
Squamous cell carcinoma	1 (5.2%)
EGFR status	
Exon 19 deletion	8 (42.1%)
L858R	2 (10.5%)
G719X	1 (5.2%)
T790M	1 (5.2%)
Wild type	7 (36.8%)
Presence of pleural effusion	
At diagnosis	17 (89.5%)
Following disease progression	2 (10.5%)
Malignant pleural effusion	15 (79%)
Target therapy	
Afatinib	4 (21%)
Erlotinib	7 (36.8%)
Osimertinib	1 (5%)
Bevacizumab	9 (47.3%)

## Supplementary Materials

The following are available online at https://www.mdpi.com/article/10.3390/biomedicines9101327/s1, Supplement Table S1: Characteristic of lung adenocarcinoma patients with MPE (*n* = 597). Supplementary Figure S1. Immunostaining for EGFR in pleural tissue of lung squamous carcinoma at a magnification of ×400. Supplementary Figure S2. Gefitinib treatment alters endothelial proliferation induced by MAPF. Supplementary Figure S3. Effects of gefitinib, bevacizumab and MAPF on endothelial permeability measured by Transwell permeability assays after 18 h treatment.
