# Supplementary material for: Concurrent Blockade of Endothelial EGFR and VEGF Signaling on Malignant Associated Pleural Fluid Induced Angiogenesis: From Clinic to Bench
Source: Biomedicines. 2021 Sep 26;9(10):1327. doi: 10.3390/biomedicines9101327 (PMC8533568; doi:10.3390/biomedicines9101327)
Supplement: Supplementary file 1 [file biomedicines-09-01327-s001.zip › biomedicines-1367129-supplementary.pdf]

Supplementary Table S1. Characteristics of lung adenocarcinoma patients with MPE (n=597).

Variable	With Bevacizumab (n=67)	Without Bevacizumab (n=530)	*p*
Gender, n(%)			0.35
Male	26 (38.8)	237 (44.7)	
Female	41 (61.2)	293 (55.3)	
Age, yr			<.0001
Median	59	68	
Range	31-91	30-105	
ECOG PS, n(%)			0.0387
0	30 (44.8)	178 (33.6)	
1	33 (49.3)	264 (49.8)	
2+	4 (6.0)	88 (16.6)	
Smoking, n(%)			
Current or former	16 (23.9)	165 (31.1)	0.22
CCI, n(%)			0.08
0	45 (67.2)	342 (64.5)	
1-2	16 (23.9)	89 (16.8)	
3+	6 (9.0)	99 (18.7)	
EGFR mutation positive, n(%)			0.38
L858R or exon19del	47 (70.1)	343 (64.7)	
Others	20 (29.9)	187 (35.3)	
EGFR target therapy drug, n(%)			
Gefitinib	23 (34.3)	241 (45.5)	0.08
Erlotinib	53 (79.1)	278 (52.5)	<.0001
Afatinib	14 (20.9)	70 (13.2)	0.09
Osimertinib	14 (20.9)	26 (4.9)	<.0001

Abbreviations: CCI, Charlson comorbidity index; ECOG PS, Eastern Cooperative Oncology Group performance status; EGFR, epidermal growth factor receptor; n, number of patients.

(A)

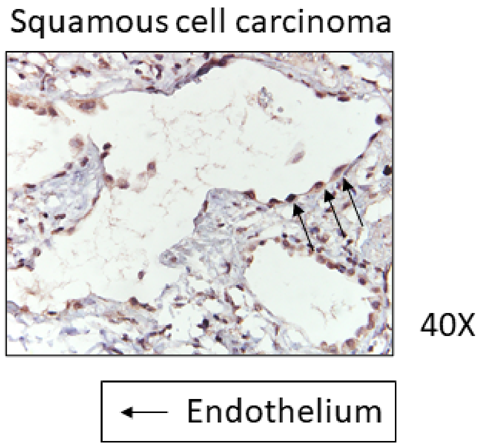

Supplementary Figure S1. Immunostaining for EGFR in pleural tissue of lung squamous carcinoma at a magnification of x400**.** Increased EGFR expression was found in the pleural endothelium of squamous cell carcinoma endothelial cells. (←, endothelial cell).

(A)

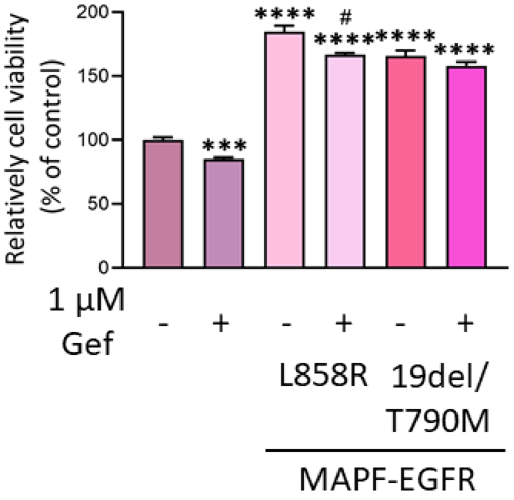

Supplementary Figure S2. Gefitinib treatment alters endothelial proliferation induced by MAPF: (A) HUVECs were incubated in the presence or absence of 1 μM gefitinib, EGFR inhibitor, and with MAPF for the indicated time. Cell viability was analyzed by the MTT assay after 24 h of treatment. ***p < 0.005; ****p < 0.0001, compared to the control group. #p < 0.05, compared to the MAPF group.

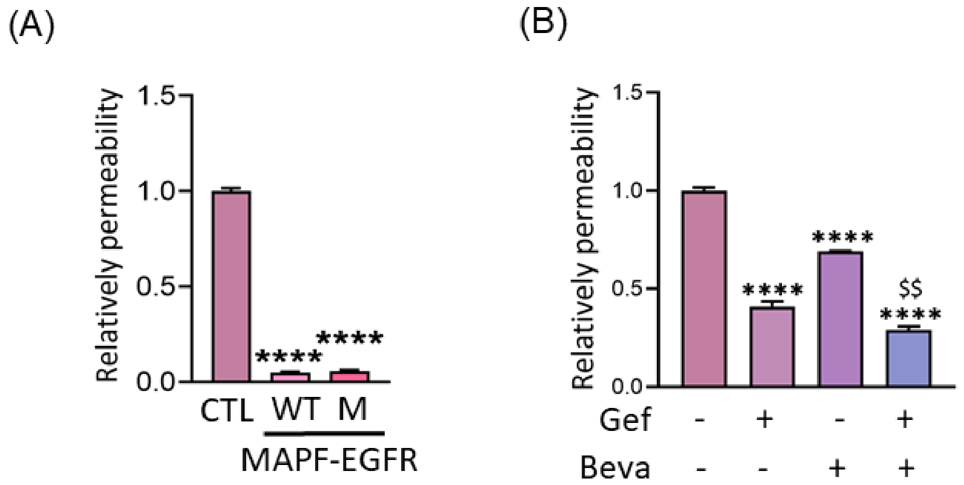

Supplementary Figure S3. Effects of gefitinib, bevacizumab, and MAPF on endothelial permeability measured by Transwell permeability assays after 18 h treatment: (A) HUVECs incubated with MAPF or control medium for 18 h. MAPF from patients with wild-type (WT) and mutant (M) EGFR were analyzed separately; (B) HUVEC cells were treated with gefitinib (Gef), bevacizumab (Beva), or Gef/Beva in combination. ****; p < 0.0001, compared to the control group. $$p < 0.01, compared to the gefitinib-treated group.
